# Challenges of an Autonomous Wildfire Geolocation System Based on Synthetic Vision Technology

**DOI:** 10.3390/s18113631

**Published:** 2018-10-25

**Authors:** Victor Arana-Pulido, Francisco Cabrera-Almeida, Javier Perez-Mato, B. Pablo Dorta-Naranjo, Silvia Hernandez-Rodriguez, Eugenio Jimenez-Yguacel

**Affiliations:** Instituto para el Desarrollo Tecnologico y la Innovacion en Comunicaciones (IDeTIC) Universidad de Las Palmas de Gran Canaria (ULPGC), 35017 Las Palmas de Gran Canaria, Spain; francisco.cabrera@ulpgc.es (F.C.-A.); jperez@idetic.eu (J.P.-M.); pablo.dortanaranjo@ulpgc.es (B.P.D.-N.); shernandez@idetic.eu (S.H.-R.); eugenio.jimenez@ulpgc.es (E.J.-Y.)

**Keywords:** thermography, infrared, geolocation, wildfire, sensor networks, synthetic vision

## Abstract

Thermographic imaging has been the preferred technology for the detection and tracking of wildfires for many years. Thermographic cameras provide some very important advantages, such as the ability to remotely detect hotspots which could potentially turn into wildfires if the appropriate conditions are met. Also, they can serve as a key preventive method, especially when the 30-30-30 rule is met, which describes a situation where the ambient temperature is higher than 30 ${}^{\circ }$C, the relative humidity is lower than 30%, and the wind speed is higher than 30 km/h. Under these circumstances, the likelihood of a wildfire outburst is quite high, and its effects can be catastrophic due to the high-speed winds and dry conditions. If this sort of scenario actually occurs, every possible technological advantage shall be used by firefighting teams to enable the rapid and efficient coordination of their response teams and to control the wildfire following a safe and well-planned strategy. However, most of the early detection methods for wildfires, such as the aforementioned thermographic cameras, lack a sufficient level of automation and usually rely on human interaction, imposing high degrees of subjectivity and latency. This is especially critical when a high volume of data is required in real time to correctly support decision-making scenarios during the wildfire suppression tasks. The present paper addresses this situation by analyzing the challenges faced by a fully autonomous wildfire detection and a tracking system containing a fully automated wildfire georeferencing system based on synthetic vision technology. Such a tool would provide firefighting teams with a solution capable of continuously surveilling a particular area and completely autonomously identifying and providing georeferenced information on current or potential wildfires in real time.

## 1. Introduction

Wildfires are one of the main reasons behind the devastation of extremely large rural and wildland areas. They also pose a very significant threat to both humans and wildlife. Unfortunately, the vast majority of these wildfires are still caused by human actions, either accidentally or intentionally [1,2,3,4].

Being able to promptly detect the occurrence of a wildfire and having the capability to then perform an accurate, real-time tracking of its evolution is the ultimate goal of firefighting teams. However, most of the methods and techniques currently used to achieve this goal rely very largely on human judgment and interaction [5,6]. For instance, most of the early detection tools used, such as thermographic imaging cameras, usually rely on a human operator to analyze a series of images acquired by thermographic cameras installed on fixed watchtowers and to make a decision based on those images [7,8].

Most of these thermographic observation watchtowers often trigger an alarm when a temperature above a certain threshold is detected, but usually there are no further automation or image processing techniques available to complement these observations and further automate the process of accurately locating the wildfire. It must also be noted that, very often, the calibration of these thermographic cameras has not been optimized for the area where it is being used, which can potentially increase the chance of having false alarms or experiencing false positive detection [9]. Once the alarm is received by an emergency dispatching facility, an operator is typically presented with the thermographic image and needs to manually assess the location of the fire based on the physical location of the watchtower and the direction from which the image was taken.

Some proposals have been made to implement either mobile or fixed sensor networks to detect the occurrence of wildfires [10,11,12], but they usually lack a certain degree of dynamic reconfigurability and ease of use. Furthermore, an emphasis was made in [13] to use low-cost components in order to make the solution more cost-effective and, therefore, more replicable.

The process described in the previous paragraph falls quite short of the automatic functionality initially envisaged, and it adds a very high degree of subjectivity, as it mainly relies on the judgment of human operators. Furthermore, wildfires can rapidly fall out of the detectable range of a fixed watchtower and, therefore, the existing infrastructure is rendered no longer useful for tracking the wildfire evolution.

A system capable of solving these limitations was implemented in [13]. It relies on an innovative use of synthetic vision technology, combined with a fully automated and rapidly deployable thermographic camera to detect and track the evolution of a wildfire in a completely autonomous manner (RDMU: Rapidly Deployable Mobile Unit). Furthermore, this system can be resiliently operated and accessed remotely for data retrieval over a series of telecommunications networks, should any of them cease to function during an emergency situation.

The way by which synthetic vision is used within this system also allows for a much better radiometric calibration of thermographic images, as atmospheric transmissivity correction is applied on a pixel-by-pixel basis based on depth mapping instead of assuming a uniform calibration of the whole thermographic image, which could potentially induce erroneous measurements if the distance difference between the foreground and background is high. This has the added benefit of generating thermographic images with a higher dynamic range that could potentially help identify much more subtle hotspots from greater distances before they develop into wildfires.

Such a system faces a series of inherent challenges affecting the reliability and accuracy of the provided geolocation data in several ways, especially regarding unit-level errors and other intrinsic errors related to the synthetic vision algorithm and how it is applied. The challenges faced during the development of this technology are discussed in this paper, and possible solutions are explored, contributing altogether to increase the overall robustness and reliability of the final system.

## 2. Sources of Error and Limitations of an Automated Wildfire Georeferencing System

The precise determination of the location (latitude, longitude, and altitude) and the accurate calculation of the attitude by the thermographic camera are the key requirements to achieving successful georeferencing. As described in [13], the generation of the synthetic image relies solely on these two sets of inputs, so a high degree of precision for their determination benefits the whole system. Also, it must account for possible errors in calibration, especially regarding radiometric effects and the possible attenuation of thermal radiation by the atmosphere, which could cause both false negatives and false positives during the fire identification process.

Within this section, we explore the main sources of error that were identified during both the development of the prototype system and during a set of functional validation field tests. A brief discussion follows that analyzes how these errors could be potentially be overcome or diminished.

**Figure 1:**
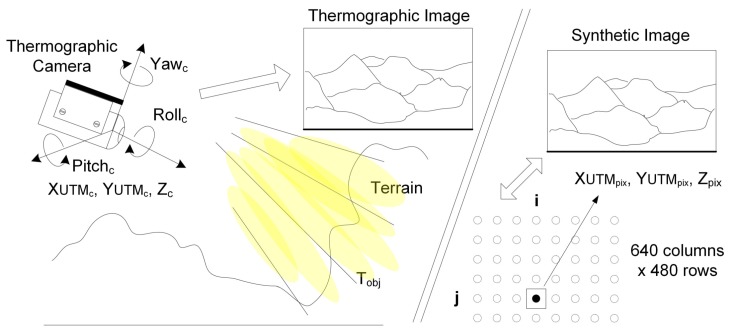
Synthetic image generation using ray-tracing techniques and optical equivalence with the thermographic image.

Figure 1 describes how the synthetic image is created in the RDMU using the ray-tracing techniques described in [13]. This process uses as inputs the thermographic camera’s location, attitude, and optical characteristics. The generated synthetic image should be optically equivalent to the thermographic image. Each pixel of the synthetic image contains latitude, longitude, and altitude information of each particular terrain point falling within the Field of View (FOV). As both the synthetic and thermographic images have been generated using the same FOV, resolution, and overall optical characteristics, each pixel in the two images can be individually cross-referenced between them.

### 2.1. Distance to the Wildfire and Temperature Threshold Determination

Once the generated synthetic image is optically equivalent to the thermographic image on a pixel-to-pixel basis, it is then possible to determine the temperature of each pixel and its geographical coordinates. The perceived temperature value for any given pixel is directly related to the distance between the camera and the geographical location of the pixel itself ($D_{pix}$), which can be computed by using Equation (1). $X_{UTMcam}$, $Y_{UTMcam}$, and $Z_{cam}$ correspond to the UTM latitude, longitude, and altitude values of the thermographic camera, and $X_{UTMpix}$, $Y_{UTMpix}$, and $Z_{pix}$ refer to the UTM latitude, longitude, and altitude of each individual pixel of the synthetic image. This distance is needed to perform a more accurate temperature determination by means of pixel-level atmospheric transmissivity calibration.

(1) $$D_{pix}=\sqrt{{\left(X_{UTMpix}-X_{UTMcam}\right)}^{2}+{\left(Y_{UTMpix}Y_{UTMcam}\right)}^{2}+{\left(Z_{pix}-Z_{cam}\right)}^{2}}$$

#### 2.1.1. Temperature Calculation

Any object subject to a given temperature $T_{obj}$ emits an amount of thermal energy $W_{obj}$ directly proportional to the fourth power of its temperature, which, according to the Stefan–Boltzmann law, can be expressed as $W_{obj}\propto T_{obj}^{4}$. However, the thermal energy received at the camera $W_{cam}$, given in $W/m^{2}$, depends mainly on the parameters described in Equation (2). The most important parameters are defined hereafter. Other parameters are dependent on the camera itself and need to be calibrated in advance [14].

- $\epsilon_{obj}$: Emissivity of the object.
- $\epsilon_{amb}$: Ambient emissivity where the object is located.
- $T_{amb}$: Ambient temperature where the object is located.
- $\tau_{atm}$: Atmospheric transmissivity between the object and the camera.
- $T_{atm}$: Atmospheric temperature considered between the object and the camera.

(2) $$\begin{array}{cc} W_{source} & =\epsilon_{obj}\cdot W\left(T_{obj}\right)+\left(1-\epsilon_{obj}\right)\cdot \epsilon_{amb}\cdot W\left(T_{amb}\right)\;\left[W/m^{2}\right]\\ W_{cam} & =\tau_{atm}\cdot W_{source}+\left(1-\tau_{atm}\cdot W\left(T_{atm}\right)\right)\;\left[W/m^{2}\right] \end{array}$$

If the thermal energy which reaches the camera is known for each individual pixel of the thermographic image, and if the remaining parameters can either be determined by calibration or reliably estimated through other means, then the temperature of each pixel can be determined with a high level of accuracy. However, it must be noted that if the estimation of these parameters is not good enough, this could induce an error in the computation of the real temperature for a given pixel and create false alarms, or, even worse, it could create a false negative and not detect a fire because its computed temperature falls below the established threshold.

#### 2.1.2. False Alarms and False Negatives

An object’s emissivity is one of the most important parameters when trying to correctly calculate its temperature by the method described in the previous section. The emissivity of an object can vary between 0 and 1 and, for the particular case of forest vegetation, it usually lies at approximately 0.8 [15]. This value, however, becomes closer to 1 if the vegetation is on fire, as burnt wood presents a higher emissivity.

Another important parameter directly related to the distance between the camera and the wildfire is the atmosphere’s transmissivity. This parameter depends on the atmosphere’s temperature and relative humidity [16] and can vary between 0.2 and 1 for distances of 5 km or less. As the distance increases, the atmosphere’s transmissivity decreases. In the particular case of the generated thermographic and synthetic images for a given terrain, the variation in distance between the camera and the location of the individual pixels can be very large across the image, so, applying a uniform distance computation to the whole image is not recommended. This is a key difference with respect to other fire detection systems, which generally apply a uniform value for the atmospheric transmissivity to the whole image and can yield erroneous temperature computations for specific areas of the image.

For instance, let us assume that we select a fire detection threshold of 200 ${}^{\circ }$C and that it will be used as a decision criterion for determining the presence or absence of a fire. If a given object is at a temperature of 100 ${}^{\circ }$C, depending on the distance, we assume that between the camera and the object we could be committing an error in the determination of the object’s temperature due to the effect of atmospheric transmissivity. For instance, if the distance is calculated to be greater than the actual distance (low atmospheric transmissivity), then the object’s temperature could end up being determined as higher than 200 ${}^{\circ }$C due to temperature overcompensation, and this would generate a false alarm. However, if we assume that the temperature of the object is now 300 ${}^{\circ }$C and we compute the distance to be less than the real one (high atmospheric transmissivity), the object’s temperature would be perceived as being lower than 200 ${}^{\circ }$C due to temperature subcompensation, thereby producing a dangerous situation where the wildfire remains undetected.

Atmospheric transmissivity curves are nonlinear and become steeper for lower values of atmospheric transmissivity, resembling a saturation-style response. This means that trying to compute the atmospheric transmissivity for distant objects (a low value of $\tau_{atm}$) becomes critical, as any subtle variation in the determination of $\tau_{atm}$ could very likely lead to a false negative and result in a possible wildfire not being detected. False negatives are always more dangerous than false alarms.

In order to prevent such high errors and risky situations, the distance from the thermographic camera to each individual pixel is computed by using a synthetic image, and then each obtained distance is used to compute an atmospheric transmissivity value for each individual pixel. Therefore, a much more precise temperature determination is created and is directly linked to the distance depth mapping. Other radiometric parameters, such as object emissivity, could be determined to further increase the precision of temperature determination. This could be achieved using the appropriate GIS layers and accessing them based on the geographical information contained in the synthetic image.

### 2.2. Optical Equivalence between Synthetic and Thermographic Images

The precision achieved when computing the distance from each of the camera’s pixels to its actual geographical location depends on both the accuracy of the generated synthetic image and the actual thermographic camera’s attitude, and how accurately the latter can be determined. This section describes how the errors associated with these processes can affect the final georeferencing of the wildfire.

#### 2.2.1. Thermographic Camera Resolution

Assuming that the optics of the thermographic camera do not introduce any distortions or that they are negligible in a far-field situation, then the minimum possible error the georeferencing system could have at a given distance in terms of ability to detect a wildfire depends on the camera’s resolution, sensor size, and focal distance. It must be clarified that, in this particular section, we are discussing how the camera’s optical parameters affect the detection of a wildfire based on its temperature, not referring to the precision in the geolocation process.

A thermographic camera’s Field of View (FOV) is determined by its lens. In the scope of this project, a thermographic camera with a focal length of 24.6 mm was used, providing an FOV of 25${}^{\circ }$ × 18.8${}^{\circ }$. However, there is another parameter which is much more interesting than the FOV and allows us to determine the minimum operating distance of the thermographic camera. This parameter is called the Instantaneous Field of View (IFOV) and accounts for the FOV covered by each individual pixel. The IFOV of a given camera is determined by its FOV, which, as we said before, is inherently linked to its optics, and also by its resolution. For this particular case, the camera used had a resolution of 640 × 480 pixels, providing an IFOV of 0.683 mrad.

The area of terrain covered by each individual pixel is, therefore, determined by the IFOV and the geographical distance from the camera to the target. This can be determined by using Equation (3), where $D_{pix}$ stands for the distance between a particular pixel and the target in meters, $A_{pix}$ is the area of terrain covered by a single pixel, and $\alpha_{IFOV}$ is the IFOV angle in radians. If we assume that we would like each individual pixel to be able to detect an area of fire of 1 m${}^{2}$, then the thermographic camera should be at a maximum distance of 1464 m from the wildfire.

(3) $$A_{pix}={\left(D_{pix}\cdot \sin\left(\alpha_{IFOV}\right)\right)}^{2}$$

However, in most cases, being able to successfully detect a wildfire or hotspot based on the information given by a single pixel is not enough and can lead to significant errors. In this case, the Johnson criteria [17] determine that at least 1.5 pixels should be used to detect any feature, meaning that we need to effectively multiply our IFOV by a factor of 1.5. This affects the maximum operational distance of the thermographic camera, and using the same example as before, the camera needs to be located at a maximum distance of 976 m to be able to detect a hotspot of 1 m${}^{2}$ based on Johnson’s criteria.

In general, this means that the maximum operational distance ($D_{pixmax}$) of the thermographic camera can be determined by the area of the smallest detectable feature desired and, therefore, can be expressed as shown in Equation (4).

(4) $$D_{pixmax}=\frac{\sqrt{A_{feature}}}{\sin(\alpha_{IFOV})}$$

In this particular case, it must be noted that we are assuming that the burnt area is completely perpendicular to the thermographic camera. However, if the terrain is at a slanted angle, then the maximum operational distance must be corrected by applying the following corrective factor. The angle $\alpha_{inc}$ is considered the incident angle and, for the fully perpendicular case, this angle has a value of 90${}^{\circ }$.

(5) $$D_{pixmax}=\frac{\sqrt{A_{feature}}}{\sin(\alpha_{IFOV})}\cdot \sin\left(\alpha_{inc}\right)$$

#### 2.2.2. Digital Elevation Model (DEM) Resolution Errors

The resolution of the DEM file, which is used as an input for the creation of the synthetic image, can also induce errors during the georeferencing phase. For instance, most DEM files are based on a 5 m grid, meaning that we are not able to determine the altitude of any object or terrain portion smaller than 5 m. During the georeferencing process, this translates into an error of 5 m added for each pixel if there is an incorrect alignment between the thermographic and synthetic images. However, due to the average dimensions of wildfires, this error can be considered low enough to still correctly detect and track a wildfire.

It must also be noted that if this type of error can be assumed as safe for the intended application, a lower DEM resolution speeds up the generation process of the synthetic image, so the improvement in the overall performance is much more desirable than the error that it has actually been committed. If higher resolutions are still required in sacrifice of computing speed, other types of DEM files based satellite observation could be used [18], which would enhance the resolution to 2 m.

#### 2.2.3. Inertial Measurement Unit (IMU) Attitude Determination Errors

The attitude of the thermographic camera is determined by means of an Inertial Measurement Unit (IMU). For this particular application, an IMU is attached to the housing of the thermographic camera and aligned with its optical axis. IMUs typically contain an accelerometer, a gyroscope, and a magnetometer to compute the attitude of the IMU unit with respect to an inertial frame, which, in this case, is the Earth.

By combining the output of each previous element, an IMU computes its spatial attitude in terms of three rotational components, called the pitch, roll, and yaw. Pitch and roll are measured with respect to the body frame of the IMU itself, but the yaw component is measured in reference to the Earth’s magnetic north, where an angle of Yaw = 0${}^{\circ }$ means that the IMU is aligned with the magnetic north.

In order to correctly generate a synthetic image that is optically equivalent to the thermographic image, the attitude of the thermographic camera needs to be precisely determined, as well as its GPS coordinates and altitude. Any error in these input parameters incurs a lack of alignment between the synthetic and thermographic images and, therefore, results in considerable errors during the automated georeferencing process.

The error introduced due to the IMU depends on the error during the calculation of each rotational component (pitch, roll, and yaw) and the distance from the thermographic camera to the wildfire. For instance, an error of $\pm 0.5^{\circ }$ in the determination of any rotational angle induces a georeferencing error of 5 m at a distance of 573 m. However, as discussed in the previous section, an error of 5 m in the georeferencing of a wildfire is almost negligible and comparable with the error already introduced by the DEM map itself. If more precision is necessary, higher-end IMUs can be used, but this unnecessarily increases the overall cost of the solution without providing a very notable difference in terms of georeferencing accuracy.

Magnetic interferences suffered by the IMU’s magnetometer are another important source of error that can increase the uncertainty in the determination of the yaw rotational component [19]. The magnetometer within the IMU is affected by distortions which are commonly known as hard and soft iron distortions. As the thermographic camera can be deployed in almost any type of terrain or location, nearby metallic structures or buildings can contribute to this effect and distort the sensed magnetic field, which ideally should only be due to the Earth’s own magnetic field. An effective procedure [20,21] for the magnetometer is imperative to overcome these magnetic interferences and must be carried out periodically to ensure the health of the sensor.

By taking into account the previous sources of error and how they contribute to wildfire georeferencing, a thorough magnetometer calibration was made. In combination with yaw angle averaging techniques, it was possible to decrease the yaw determination error to $\pm 0.2^{\circ }$, which means that a georeferencing error of 20 m occurs at a distance from the wildfire of 5.7 km, which is quite acceptable when taking into account the intended detection range.

#### 2.2.4. GPS Altitude Determination Errors

The vertical component (Z-axis or altitude) of a GPS fix is between 2 and 5 times less accurate than the horizontal components (X and Y or longitude and latitude) [22]. In order to achieve the best possible accuracy when determining a GPS fix, the satellites in view should be spaced apart as much as possible, ideally 120${}^{\circ }$ from each other, but this ideal condition is not always achieved. At least three satellites are needed to achieve a 2D fix, and four (including altitude information) are required to achieve a 3D fix.

A 2D fix from a GPS can be usually considered sufficiently good when the Horizontal Dilution of Precision (HDOP) reported by the GPS unit presents a value lower than 1. A value below this threshold can be typically achieved after the GPS unit has been on for a few minutes, has been computing the actual location, and has converged to a stable solution in both latitude and longitude.

In order to obtain the same accuracy for the vertical component as the horizontal ones, the satellites used to compute the fix should also be spaced 120${}^{\circ }$ perpendicular to the receiver. However, this is not possible, as the terrain itself will always interfere with at least one of the satellites. Even if we considered a completely flat terrain, only two satellites would be available within the 120${}^{\circ }$ ideal separation span. Furthermore, even if the satellites were closer together, they would be too close to the horizon, where propagation conditions are far from ideal and cause additional errors in the signals, thus incurring further altitude determination inaccuracy.

In addition to the previous limitation, GPS receivers use a WGS-84 ellipsoid [23] to determine the reported altitude. The WGS-84 standard is based on a theoretical ellipsoid which, at some points, falls far from the real topographical altitude of a given point. This renders the vertical component almost completely unreliable for achieving a precise synthetic image of the terrain.

To overcome this issue, the implemented system takes only the 2D solution of the GPS when HDOP < 1, and then it obtains the altitude component from the locally stored DEM map.

## 3. Quantization of IMU/GPS-Induced Errors

As part of the full system’s validation, it is necessary to empirically quantify the misalignment that potentially exists between the synthetic and thermographic images due to the previously discussed sources of error. This was achieved by generating a series of synthetic images based on both the manually specified parameters and the inputs taken directly from the IMU and GPS, and then comparing the generated synthetic images with known topographical features or landmarks to establish the error obtained. This error was evaluated by determining the pitch, roll, and yaw offset angles that were to be applied to the measured ones so that the synthetic image was fully aligned with the known topographical features.

In all cases, the thermographic camera’s attitude (pitch, roll, and yaw angles) was determined using an IMU attached to the camera body, and only after applying the magnetometer calibrations previously discussed. The location coordinates were obtained using a high-precision GPS receiver when HDOP < 1. The vertical component or altitude provided by the GPS was discarded and substituted by the one obtained from the DEM map used to generate the synthetic image.

**Figure 2:**
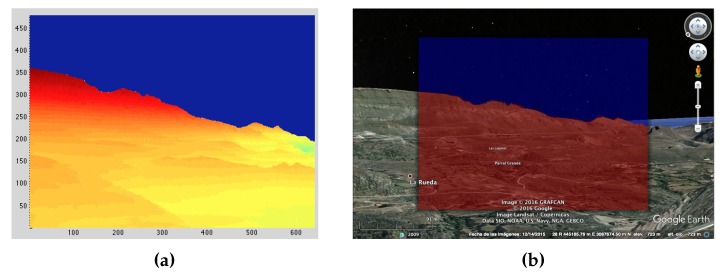
Synthetic image alignment test with known landmarks. (**a**) Synthetic image after manually applied pitch, roll, and yaw corrections. (**b**) Synthetic image superimposed on Google Earth.

Figure 2 shows the generated synthetic image based on the following input parameters: Longitude = −15.5571${}^{\circ }$, Latitude = 27.9129${}^{\circ }$, Altitude = 723 m ASL, Yaw = 126.30${}^{\circ }$, Pitch = −0.94${}^{\circ }$, and Roll = −3.06${}^{\circ }$. It must be noted that these parameters were manually specified and did not come from GPS or IMU data. An FOV of 25${}^{\circ }$× 18.8${}^{\circ }$ was used to generate the image, which matches the optical characteristics of the thermographic camera’s lens. For comparison, the synthetic image is superimposed with the one obtained by applying those same input parameters to Google Earth.

The pitch angle just had to be manually corrected by an additional 0.06${}^{\circ }$ in order to make both images equivalent. This means that a georeferencing error of 5.2 m would have occurred at a distance of 5 km, which indeed matches the resolution of the DEM model used and confirms that the synthetic image generation works as expected and is limited by the DEM model resolution in an ideal case. This means that any error observed from this moment onward is not due to the synthetic image generation process, but due to IMU or GPS data inaccuracies.

A second test was then carried out, but, in this case, it was obtained using the pitch, roll, and yaw parameters as indicated by the IMU. This time, the yaw angle had to be manually corrected by 1.01${}^{\circ }$ in order to make both images equivalent. This means that a georeferencing error of 54.37 m would have occurred at a distance of 3 km, which is the greatest distance in the synthetic image. In this case, we are observing an additional error due to the IMU inaccuracies, which demands an attitude error correction.

**Figure 3:**
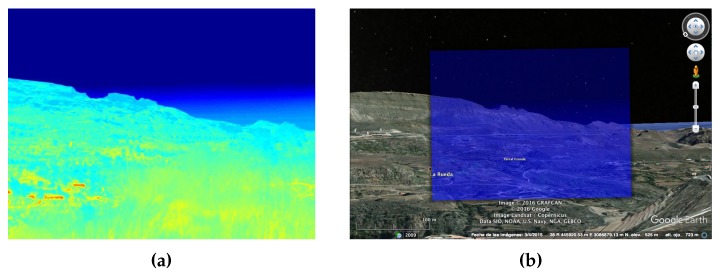
Thermographic image equivalent to the synthetic image after attitude compensation. (**a**) Thermographic image. (**b**) Thermographic image superimposed on Google Earth.

Once the attitude errors were computed for the synthetic image, the thermographic image could then be superimposed directly over the terrain, as they were now equivalent to each other in terms of perspective and geometry. Figure 3 shows the thermographic image superimposed over the same terrain area, proving that it now has the correct alignment and can be used to detect and georeference possible hotspots.

## 4. Terrain Profile Alignment

On several occasions, the error in terms of misalignment between the synthetic and thermographic image was too high to be deemed acceptable for georeferencing use. This was mostly due to inaccuracies of the IMU when reporting the thermographic camera’s attitude in terms of yaw, pitch, and roll angles. Also, the GPS unit contributed to this misalignment by providing inaccurate latitude and longitude coordinates, especially if the HDOP was not optimal, which is usually the case near dense forest canopy.

**Figure 4:**
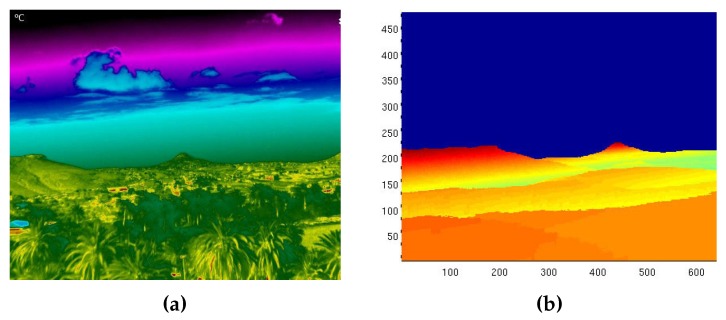
Synthetic image with large disalignment with respect to the thermographic Image. (**a**) Thermographic image. (**b**) Synthetic image.

An example of a case where both the generated synthetic image and thermographic image were not correctly aligned by a large margin can be observed in Figure 4. In this case, the error was mostly in the yaw angle and had a value of approximately 5${}^{\circ }$ if compared with known landmarks. Usually, the yaw angle is the one more prone to suffer from errors, since the magnetometer requires careful calibration in order to compensate hard and soft iron effects, and any local magnetic interference can have a very negative impact on the accuracy.

Due to this large misalignment and the fact that the synthetic image cannot be used to georeference any hotspots under these circumstances, an automated method for coping with these sorts of errors had to be developed. This paved the way for the development of the synthetic terrain alignment process described in this section. In particular, performing a terrain profile alignment by comparing the synthetic and thermographic images enables the system to automatically compute the necessary pitch, roll, and yaw offset which need to be applied to the synthetic image to compensate for any IMU or GPS errors and, therefore, to improve the optical equivalence between the two images.

Once the thermographic camera’s attitude was determined by the IMU and the GPS receiver, a new step was introduced. This step was carried out before starting the actual georeferencing process. In particular, this process attempted to actively determine the profile, or interface curve, between the terrain and sky in both the synthetic and thermographic images so that a set of error offsets could be computed and applied to the pitch, roll, and yaw angles.

**Figure 5:**
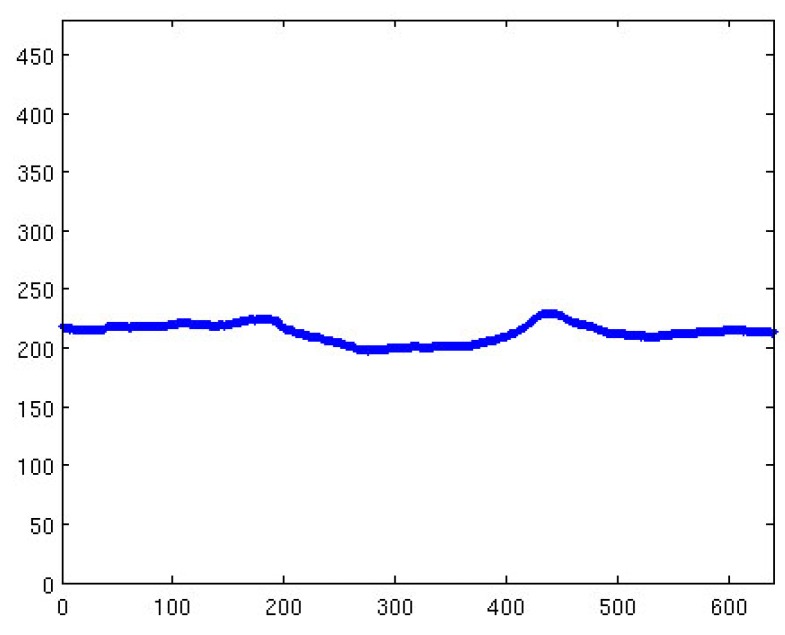
Computed sky–terrain interface using the synthetic image.

As previously discussed, the synthetic image contains the coordinates of each pixel as well as its altitude. During the generation of this synthetic image, any pixel not part of the terrain was given an altitude of zero. This was achieved during the ray-tracing process, where any ray not impacting the terrain was considered to be part of the sky. For every column in the synthetic image, a search was conducted to find any pixel immediately preceding a pixel with an altitude value of zero. Every time this occurred, the row in which this was detected was saved in a 640-element vector. This process determined the boundary between the sky and terrain in the synthetic image, as can be seen in Figure 5.

The next step in the process was to find the same information in the thermographic image; this step enabled the matching of both profiles afterward. In this case, obtaining the boundary between the sky and terrain was not as immediate, as the thermographic image contains temperature values. However, the temperature values of the sky and terrain are quite different from each other, so the location of the actual interface was determined by computing the interpixel absolute differences.

**Figure 6:**
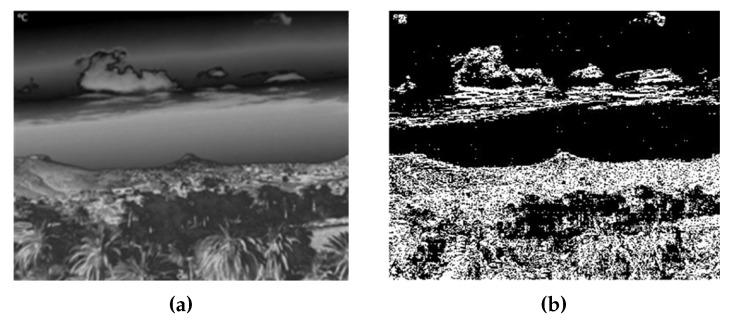
Binarized temperature difference image generation based on the thermographic image. (**a**) Thermographic image converted to 256-level grayscale. (**b**) Binarized temperature difference image.

The thermographic image was first converted into a 256-level grayscale image. From this image, a matrix containing the absolute temperature differences between each row was calculated by assuming $\left|Z_{pix}\left[j+1,i\right]-Z_{pix}\left[j,i\right]\right|$. Every value that did not fall above a given threshold (10 in this case) was assigned a value of zero. This was to limit the number of differences to those actually significant enough to be representative of a sky–terrain interface. After this, the absolute difference image was binarized, where each pixel had a value of either 1 or 0 depending on whether its temperature difference falls above or below the threshold. This new image has a dimension of 640 × 479 pixels and can be seen in Figure 6.

Once both curves were obtained, a correlation process was applied to both the synthetic and thermographic sky–terrain interface in order to compute the pitch, roll, and yaw difference to be applied to the synthetic image. This is required to render it optically equivalent to the thermographic image.

**Figure 7:**
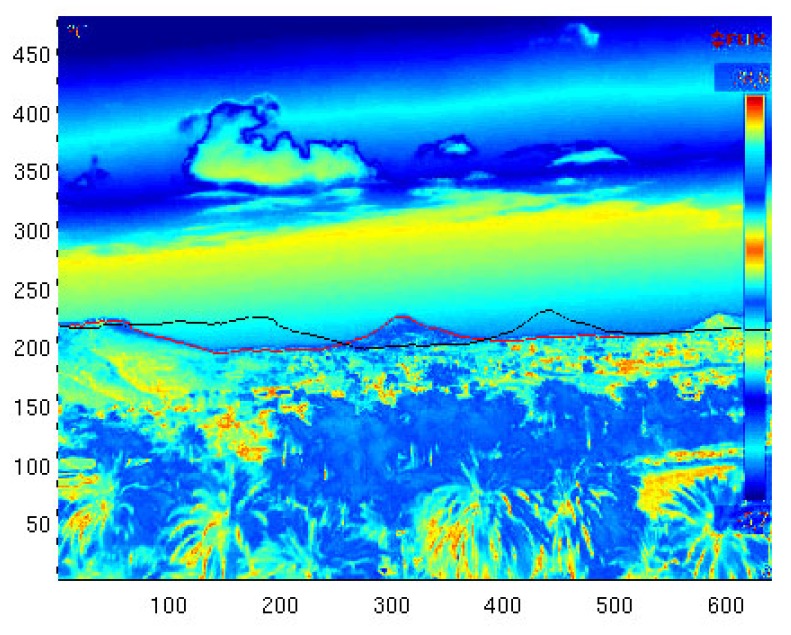
Pitch, roll, and yaw offset determination based on the sky–terrain interface.

**Figure 8:**
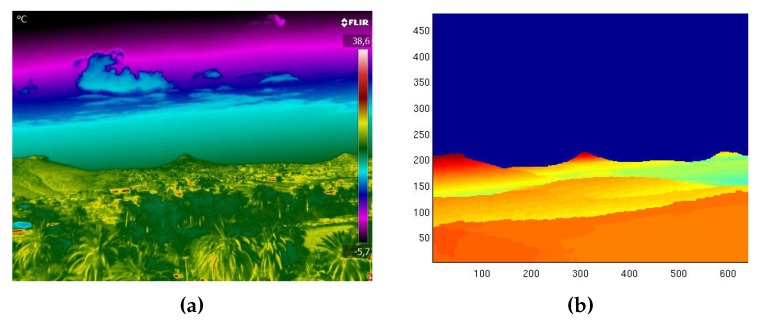
Final result and comparison of the thermographic and synthetic images after performing a terrain profile alignment. (**a**) Thermographic image. (**b**) Fully aligned synthetic image.

Figure 7 shows the output of this process and how the offset of $\Delta Yaw=5.2^{\circ }$, $\Delta Pitch=0.2^{\circ }$, and $\Delta Roll=-0.5^{\circ }$ was computed. After the offset was determined, the corrected synthetic image could then be generated, which was then the input to the wildfire georeferencing process. Once the alignment was performed, the result was as seen in Figure 8.

**Figure 9:**
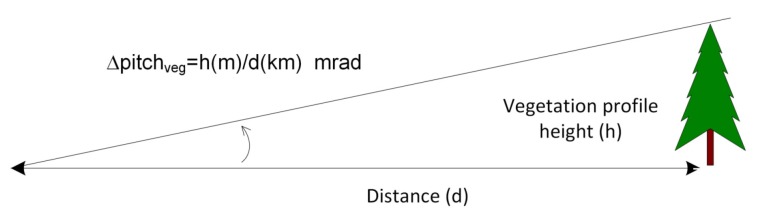
Error due to the presence of vegetation at the sky–terrain interface.

It should also be noted whether there is vegetation relatively close to the thermographic camera because it can cause errors when determining the location of the sky–terrain interface curve, as the top of the trees may be interpreted as terrain. Due to the fact that the DEM map does not contain any vegetation altitude data, this difference could be a source of error during the previously described process. Depending on the distance from the thermographic camera to the vegetation, this error was computed as an angle relative to the terrain. For instance, an error of 0.2${}^{\circ }$ would occur if the vegetation had an altitude of 3.5 m at a distance of 1 km. This error was evaluated in a generic form, as shown in Figure 9.

## 5. Field Test during Prescribed Burns

Several outdoor tests with real fire were carried out in order to evaluate the overall effectiveness of the proposed system. These tests were part of a functional validation campaign during a series of controlled burns on the island of Gran Canaria, Spain. These controlled burns were coordinated and executed by professional firefighting staff from the local council and the military emergency response unit (UME) as part of their routinary firewall maintenance. All results were then carefully analyzed in order to quantify the errors induced during the process and the overall operability of the system.

**Figure 10:**
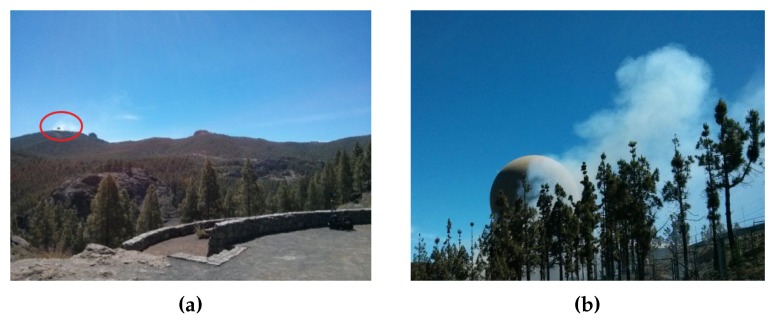
Controlled burn carried out by professional firefighters. (**a**) General view. (**b**) Close-up view.

Figure 10 shows one of the locations where these tests took place. These images serve only as an indication of the sort of environment where the system was operating. In this particular case, the RDMU was located at a distance of 2.5 km from the controlled burn.

**Figure 11:**
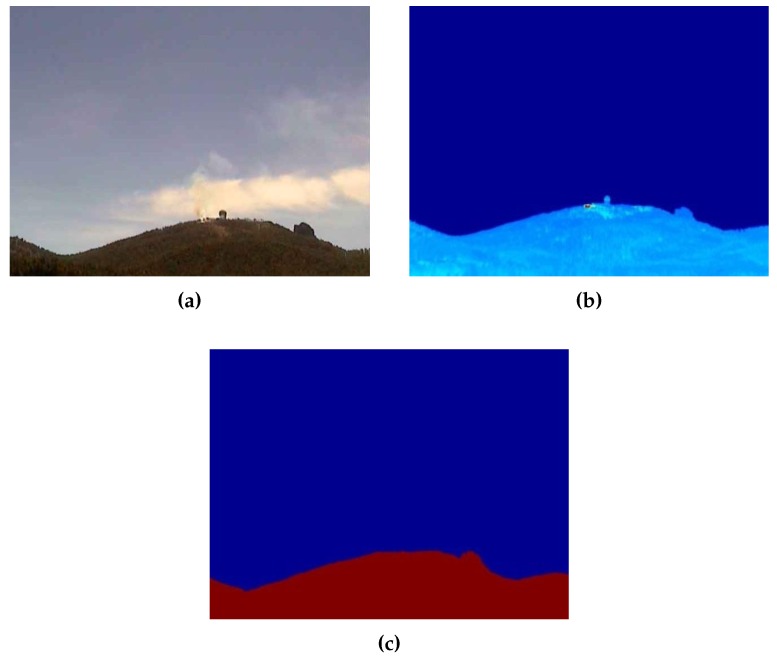
Set of images produced by the RDMU during nominal operation. (**a**) Visible channel. (**b**) Thermographic image. (**c**) Synthetic image.

**Figure 12:**
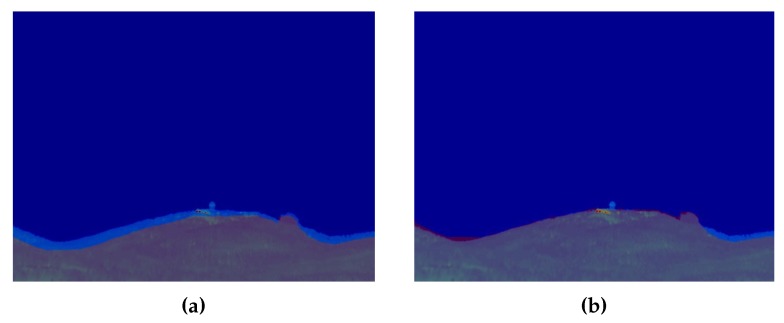
Effectiveness of the terrain profile alignment process. (**a**) Before terrain profile alignment. (**b**) After terrain profile alignment.

**Figure 13:**
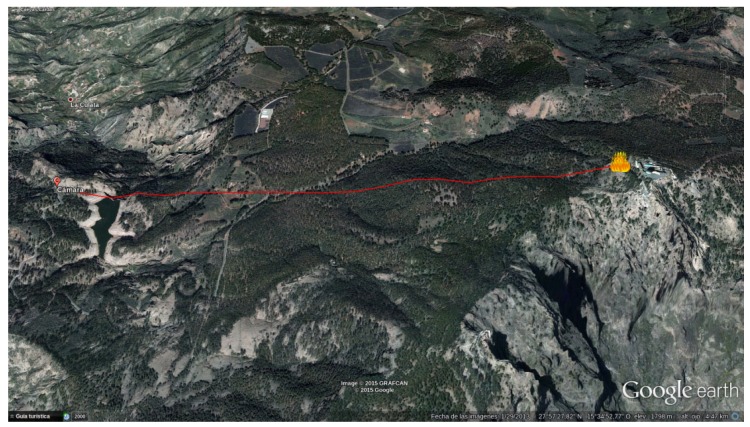
Automated geolocation of the wildfire seen with Google Earth.

The generated synthetic and thermographic images can be seen in Figure 11. After generating the synthetic image, it was fed together with the thermographic image into the terrain profile alignment process described in the previous section. This process determined that an error compensation offset of 0.99${}^{\circ }$ in the pitch angle was required, translating to an error of 44.23 m at the operational distance of 2.5 km. The georeferencing process also accounted for an additional 0.13${}^{\circ }$ due to the presence of vegetation with an estimated height of approximately 6 m. The difference before and after the terrain profile alignment process can be seen in Figure 12. The correctly georeferenced wildfire was then automatically exported to a GIS visualization tool—in this case, Google Earth—for visualization purposes, as seen in Figure 13.

The full identification and geolocation of the wildfire (in this case, represented as a prescribed burn) was demonstrated to be achievable with no human intervention at all, other than setting up the camera and powering the unit. All of these results were also relayed in real time to a nearby operations center, which had full bidirectional access to telecommand and telemetry from the whole system over a triple-redundant telecommunications networks.

**Figure 14:**
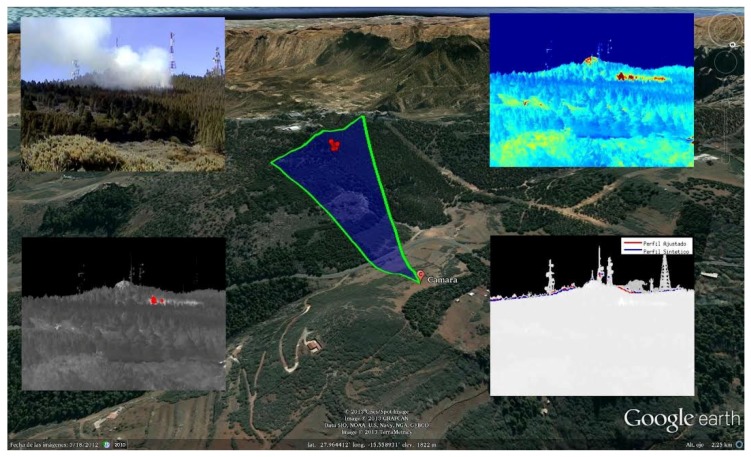
Final result of the RDMU in full autonomous operation.

The final result of the RDMU system for another controlled burn can be seen in Figure 14, which shows the full graphical representation over a GIS tool (in this case, Google Earth) as it would be seen at the emergency dispatch room during a wildfire outburst. The blue area indicates the FOV of the camera, which rotates dynamically to show where the camera is pointing at any given moment and the area of terrain currently being monitored. The red dots represent the georeferenced hotspots detected by the thermographic camera.

## 6. Discussion and Conclusions

The developed system proved to be able to achieve the objective proposed at the beginning of this paper. It was particularly demonstrated that the automatic geolocation of wildfires can be carried out by performing a data fusion of thermographic images and synthetic vision, while reducing the limitations of current solutions based on fixed observation points.

The possibility of achieving pixel-level calibration in terms of atmospheric transmissivity is a novel approach and a contribution to this research topic. As far as the state-of-the-art analysis carried out, no similar system has been identified that performs this type of calibration based on synthetic vision. This capability not only enables a much more precise calibration of the temperature perceived by a thermographic camera, but it can also yield a much broader dynamic range on the generated thermographic images. All studied thermographic analysis cases applied a uniform radiometric calibration to the whole image, and this has proven to be an important source of temperature determination error if the distance to the object being measured is not precisely known.

A high-precision GPS and a correctly calibrated IMU are also mandatory for the correct generation of the synthetic image. During the test campaigns, it was observed that the GPS receiver occasionally took a large amount of time to converge to the required HDOP level. This can be mitigated by storing within the GPS receiver a recent ephemeris of the GPS constellation, which enables a faster convergence. The IMU was also demonstrated to be prone to magnetic interference in several environments, especially in the vicinity of power lines or metallic structures. This causes the yaw component to usually have an error of several degrees, but it was possible to compensate for it by using the terrain profile alignment procedure. After performing some field tests with this method in place, the RDMU was able to compensate for attitude determination errors higher than 5${}^{\circ }$.

The accuracy level achieved when measuring the remote temperature of a hotspot using the thermographic camera was demonstrated to be absolutely dependent on the correct determination of the atmospheric transmissivity. If both the atmospheric temperature and relative humidity are precisely determined, the distance between the thermographic camera and the hotspot remains the main error-inducing factor. If this distance is erroneously calculated, then the chances of having false alarms or false positives are severely increased. The determination of this distance relies entirely on the alignment of both the thermographic and synthetic image, as well as the accuracy of the synthetic image. A more computationally intensive approach to the ray-tracing technique could be applied in order to improve the synthetic vision algorithm and make it more robust with respect to different terrain configurations.

Feedback from the firefighting staff is critical to fine-tune the performance of the system. Positive feedback was received highlighting that the automated georeferencing capability developed and presented in this paper served to speed up decision-making mechanisms, as well as reduced the uncertainty of the generated geographical data. The portable form factor was also highly praised due to its increased maneuverability and dynamic configuration options while performing wildfire suppression tasks.

Several field tests with prescribed burns were carried out in Spain—more precisely, in the Canary Islands (Gran Canaria, Tenerife, La Gomera, and La Palma) and in Valencia. These locations presented very different terrain configurations that served to evaluate the feasibility of the RDMU with the direct involvement of professional wildfire suppression staff and under real operational conditions.

## Abbreviations

The following abbreviations are used in this manuscript:

FOV	Field of View
IFOV	Instantaneous Field of View
DEM	Digital Elevation Model
IMU	Inertial Measurement Unit
HDOP	Horizontal Dilution of Precision
RDMU	Rapidly Deployable Mobile Unit
